# Potential Therapeutic Applications of Natural Compounds in Diabetes-Associated Periodontitis

**DOI:** 10.3390/jcm11133614

**Published:** 2022-06-22

**Authors:** Min Yee Ng, Taichen Lin, Shih-Chi Chao, Pei-Ming Chu, Cheng-Chia Yu

**Affiliations:** 1 School of Dentistry, Chung Shan Medical University, Taichung 40201, Taiwan; ngminyee_92@hotmail.com (M.Y.N.); taichenlin23@gmail.com (T.L.); 2 Department of Dentistry, Chung Shan Medical University Hospital, Taichung 40201, Taiwan; 3 Institute of Oral Sciences, Chung Shan Medical University, Taichung 40201, Taiwan; h5l4g4fu6123@gmail.com; 4 Department of Medical Research and Education, Lo-Hsu Medical Foundation, Lotung Poh-Ai Hospital, Yi-lan, Luodong 265501, Taiwan; 5 Department of Anatomy, School of Medicine, China Medical University, Taichung 404333, Taiwan; pmchu@mail.cmu.edu.tw

**Keywords:** diabetes-associated periodontitis, natural compounds

## Abstract

Diabetes mellitus (DM) is a major worldwide health burden. DM is a metabolic disease characterized by chronic hyperglycemia, and if left untreated, can lead to various complications. Individuals with uncontrolled DM are more susceptible to periodontitis due to both a hyper-inflammatory host response and an impaired immune response. Periodontitis, on the other hand, may exacerbate DM by increasing both local and systemic inflammatory components of DM-related complications. The current standard for periodontal treatment in diabetes-associated periodontitis (DP) focuses mostly on reducing bacterial load and less on controlling the excessive host response, and hence, may not be able to resolve DP completely. Over the past decade, natural compounds have emerged as an adjunct approach for modulating the host immune response with the hope of curing DP. The anti-oxidant, anti-inflammatory, and anti-diabetic characteristics of natural substances are well-known, and they can be found in regularly consumed foods and drinks, as well as plants. The pathophysiology of DP and the treatment benefits of various bioactive extracts for DP will be covered in this review.

## 1. Introduction

About 537 million people worldwide have diabetes mellitus (DM) and this number is expected to rise drastically to 643 million by 2030 and 783 million by 2045 [1]. According to WHO, DM is a chronic, metabolic disease characterized by elevated levels of blood sugar, which over time, leads to serious damage to the heart, blood vessels, eyes, kidneys, and nerves. Untreated DM also poses a major risk for periodontitis, a multifaceted local inflammatory condition of the tooth-supporting structures [2]. Periodontitis normally develops as a result of the host’s reaction to local microbial stimuli and excessive blood sugar levels contribute to the prevalence and severity of this local disease by amplifying the host response [3]. Periodontitis is mainly characterized by periodontal pocket formation, loss of connective tissue attachment, alveolar bone resorption, and if left untreated, can lead to tooth loss [4]. Some studies have revealed that an altered host immune response in DM results in excessive inflammation and increases the severity of periodontal tissue destruction in diabetes-associated periodontitis (DP) [5,6]. In fact, patients with DP exhibit greater alveolar bone loss and a poorer prognosis after standard treatments compared with patients who do not have DM [5].

At times, conventional methods, such as mechanical scaling and root planing (SRP) and antiseptic mouthwash, may not be able to resolve DP completely. SRP involves mechanical removal of the bacterial biofilm that has adhered to the tooth or root surfaces, although it may not fully eradicate bacteria, especially in difficult-to-reach spots, such as the furcation areas. Antiseptic mouthwash, such as chlorhexidine (CHX), is often used as an adjunct to periodontal therapy since it is effective against various oral microbes. However, a few studies have revealed that prolonged use of CHX can cause several adverse effects, including tooth discoloration, parotid gland swelling, and taste disturbances [7]. Aside from that, SRP is occasionally supplemented with systemic antibiotics in cases of aggressive periodontitis. Even so, repeated and frequent use of antibiotics promotes the development of bacterial resistance, which is a grave public health concern [8]. The mentioned standard periodontal treatments principally focus on reducing bacterial burden and less on controlling the excessive host response in DP. Thus, it is imperative to focus on alternative products that can regulate the host’s hyper-inflammatory state to curb DP. Over the past decade, natural compounds have emerged as an adjunct approach for modulating the host immune response in an attempt to cure DP. Natural compounds in commonly consumed foods and beverages, as well as herbs, are well-known for their anti-oxidant [9], anti-inflammatory [10], and anti-diabetic properties [11]. Although recent reviews have assessed the impact of natural compounds on diabetes [12,13,14] and periodontitis [15,16], reviews on the effect of natural product on DP are lacking. To this end, this review includes all animal and clinical studies that have reported outcomes for natural compounds in DP. To date, the natural compounds that can modulate DP are resveratrol [17,18,19,20,21], curcumin [22,23,24,25,26,27], melatonin [28,29,30,31,32], propolis [33,34,35], vitamin C [36,37,38], green tea [39,40,41], ginger [42], aloe vera [43], rutin [26], and vitamin E [44]. Thus, this review will cover the pathogenesis of DP and the therapeutic effects of the above mentioned extracts on DP.

### 1.1. Protocol and Search Strategy

The protocol for this scoping review was performed according to the guidelines of the Preferred Reporting Items for Systematic Reviews and Meta-Analyses extension for Scoping Reviews (PRISMA-ScR). The purpose of this study was to determine the impact of different natural products on DP as stated in the present literature. PubMed, Embase, and WoS databases were searched for the keywords (“natural products” OR natural compounds OR herbs OR natural medicine OR anti-oxidant) AND (“periodontitis”) AND (“diabetes”) from 2012 to the end of April 2022. The included articles were supplemented by a manual search of the bibliographies of all listed papers.

**Figure 1:**
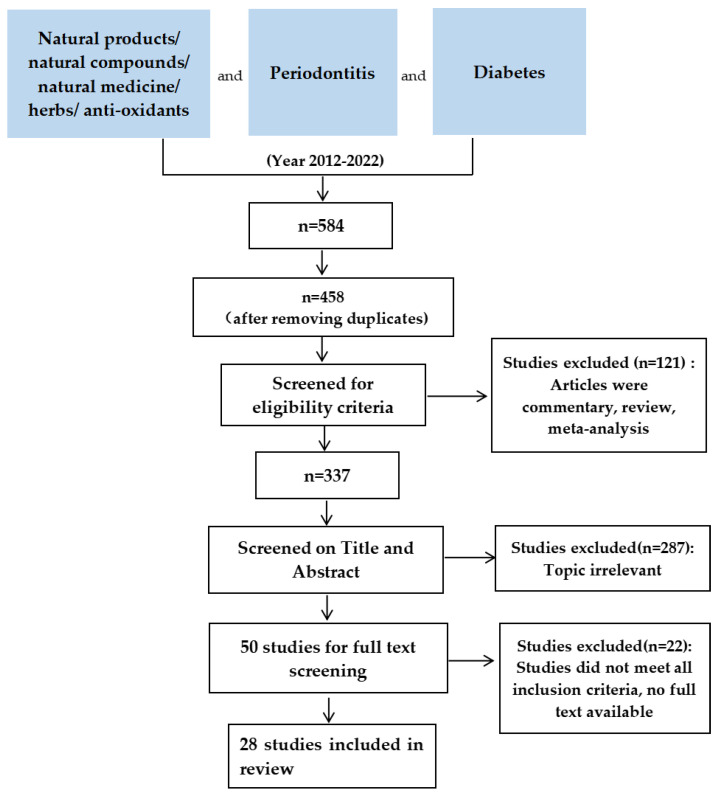
Study selection flow chart.

The inclusion criteria required studies of natural products and their effect on DP in vivo. No restrictions were placed on the language. Exclusion criteria included commentary, reviews, systematic reviews, meta-analyses, and books/book chapters. As for study selection and data extraction, the selection process involved three stages (Figure 1): (1) databases were searched, duplicate papers were manually eliminated, and the remainder screened for eligibility; (2) papers retrieved in stage 1 were screened based on titles and abstracts using the inclusion and exclusion criteria; (3) complete texts were analyzed using the same criteria. Figure 1 depicts the flow of the study selection process.

### 1.2. Pathogenesis of Diabetes-Associated Periodontitis

In diabetic patients, hyperglycemia induces production of advanced glycation end-products (AGEs) [45]. AGEs play a crucial role in the progression of DM-related complications [46], for instance, periodontitis. Periodontitis is a local inflammatory condition of the tooth and surrounding structures, such as connective tissue and alveolar bone [4]. This localized inflammatory condition is mainly triggered by the host response to pathogenic bacteria in the oral cavity, which causes the structures to break down. It was evidenced that the prevalence and severity of periodontitis is greater among diabetic subjects compared to non-diabetic patients [47,48]. In DM, the build-up of AGEs leads to increased susceptibility to periodontitis through several mechanisms [49,50]. It is thought that AGEs can alter immune cells, stimulate production of reactive oxygen species (ROS) leading to increased oxidative stress [51], and enhance the release of proinflammatory mediators [52].

The interaction between AGE and its receptor (RAGE) yields long-term effects on neutrophils, macrophages, and monocytes, which are the key cellular targets of diabetes-associated periodontitis (DP). People with DM often have enhanced neutrophils activation [52] and a shift in monocyte/macrophage phenotype [49]. Activated neutrophils trigger the production of a variety of chemokines and cytokines necessary for inflammatory and immune responses [53]. These include the promotion of cellular oxidant stress, activation of the transcription factor nuclear factor kappa B (NF-κB), and production of pro-inflammatory cytokines, such as IL-1β and TNF-α [49,54,55]. With exaggerated activation of neutrophils, proteases are released into the surrounding periodontal tissues that can cause excessive damage [56,57].

Among the downstream effects of inflammatory cytokines, the up-regulation of matrix metalloproteinases (MMPs) is controlled by activation of the transcription factor NF-κB [58,59,60]. It is well recognized that MMPs, such as the collagenases MMP-1, MMP-8, and MMP-13, and the gelatinases MMP-2 and MMP-9, are responsible for the destruction of various constituents of the extracellular matrix, including collagen [61,62]. Moreover, these MMPs produced locally by inflamed oral tissues can travel through the circulation and degrade insulin receptors. This indirectly contributes to insulin resistance, promotes hyperglycemia, and exacerbates the diabetic condition [63,64].

Oxidative stress is well recognized for its involvement in degradation of the extracellular matrix and stimulation of bone resorption by osteoclasts in periodontal tissue [65,66]. The possible sources of oxidative stress in DM include auto-oxidation of glucose, shifts in redox balances, decreased tissue concentrations of low molecular weight anti-oxidants, such as reduced glutathione (GSH), and impaired activities of anti-oxidant defense enzymes, such as superoxide dismutase (SOD) and catalase (CAT) [51]. In DM patients, the activity of NADPH oxidase (NOX) is elevated in activated neutrophils [53,67]. NOX is considered the major source of radical oxygen species (ROS) [67]. The generation of ROS by NOX, also known as the oxidative burst, results in the eradication of microbial invasion by macrophages and neutrophils, and hence functions as an inflammatory mediator [68]. Moreover, the impaired expression of anti-oxidants, which are pre-dominantly mediated by the NrF2 transcription factor, further contributes to ROS-associated damage [69].

**Figure 2:**
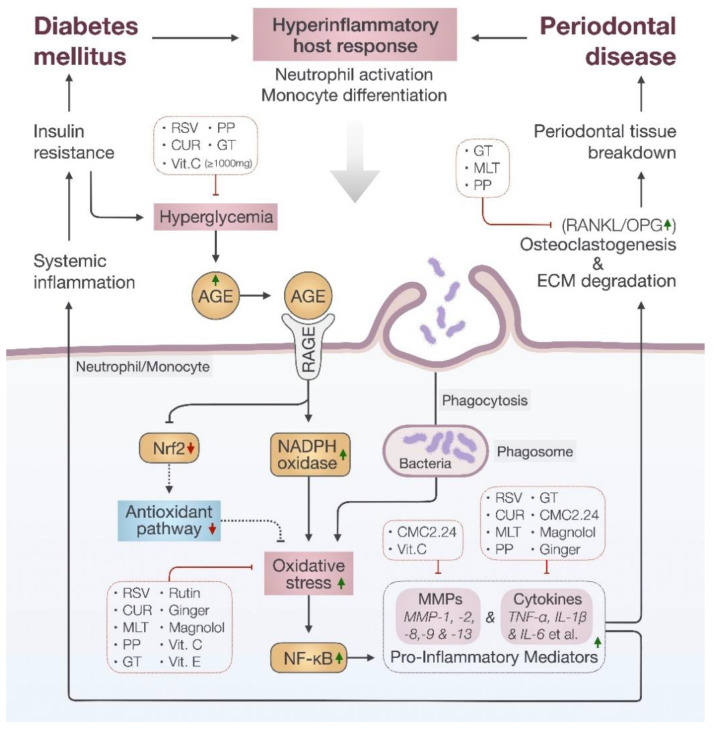
Summary of the pathogenesis of diabetes-associated periodontitis (DP).

Despite the fact that diabetes is an independent risk factor for periodontitis [70], periodontitis has a deleterious impact on the glycemic control of diabetes [71]. Diabetic patients with periodontitis may present with greater systemic inflammatory, as seen in elevated serum levels of IL-6, TNF-α, and CRP. A raised inflammatory state, on the other hand, can worsen both insulin resistance and glycemic control [72]. In a nutshell, individuals with DM appear to be more susceptible to periodontitis for two reasons: first, because of hyper-inflammatory components that lead to a predisposition for tissue destruction, and second, because of an impaired immune response, which may delay the host’s natural healing/regenerative abilities during the disease course. Periodontitis, on the other hand, might increase the diabetes-related host inflammatory component both locally and systemically, exacerbating DM. A summary of the pathogenesis of DP is illustrated in Figure 2. In the following sections, we will discuss the role of different natural compounds in modulating the host response in DP. Table 1 summarizes the study types of each natural compound and their clinical evaluation.

## 2. Therapeutic Effect of Natural Compounds in Diabetes-Associated Periodontitis

### 2.1. Resveratrol

Resveratrol (RSV) is a common stilbene present in various foods, such as grapes, peanuts, pistachio, and cranberry [73]. RSV is known for its anti-oxidant, anti-inflammatory, and even anti-cancer qualities [74,75]. The therapeutic potential of RSV for DP has been explored in three animal studies and two clinical trials, as tabulated in Table 2. All investigations agreed on the anti-inflammatory effects of RSV [18,20,21,76], and three confirmed its anti-diabetic [18,20,21] capabilities for managing DP. The results of these studies revealed a remarkable attenuation of bone loss, reduced inflammatory cytokines, and improved fasting blood glucose levels, so it can be considered an effective approach for reducing bone resorption and improving metabolic management.

Zhen et al. demonstrated the anti-inflammatory and anti-diabetic properties of RSV in experimental DP mice and murine gingival epithelial cells in vitro [21]. When RSV was administered to DP mice for 4 weeks, there was significant improvement in fasting blood glucose (FBG) and reduced alveolar bone loss compared to mice without RSV treatment. Moreover, supplementation repressed the elevated levels of TLR4 induced by DM/DP [77,78,79] as well as down-regulating inflammatory cytokine levels in both DP mice and gingival epithelial cells [76]. These findings strongly imply that RSV suppressed DP by improving hyperglycemia and the anti-inflammatory capability of the host by inhibiting both TLR4 expression and activation of its downstream signaling [80].

When Fabiano et al. compared the effect of RSV alone and RSV plus insulin (INS) on DP mice, the efficacy of RSV was comparable to insulin for reducing blood glucose levels [81]. RSV, in addition, had a synergistic effect with insulin on lowering blood glucose levels and inflammatory cytokines, such as IL-1β, IL-7, IL-6, and attenuating bone loss [81]. Furthermore, quantification of oxidative stress in biopsied gingiva from intervened DP mice revealed enhanced SIRT 1 and SOD levels, and reduced NADPH oxidase activity, similar to earlier studies [21,82]. Silent information regulator 1 (SIRT 1) [83] and superoxide dismutase 1 (SOD) are both anti-oxidative enzymes that regulate oxidative stress and protect cells from ROS toxicity [84,85].

In contrast, NADPH oxidase is an oxidative enzyme that catalyzes superoxide and peroxide generation, and hence, is responsible for the up-regulation of ROS production [86]. Since RSV has been shown to reduce the expression of NADPH oxidase in current and prior studies [87], this indicates that RSV can reduce the impact of oxidative stress mediated by ROS by quenching ROS molecules and reducing their production as well [88]. Thus, the anti-oxidant capacity of RSV is linked to its influence on SIRT 1, SOD 1, and NADPH oxidase levels, which helps to reduce the development and/or impact of oxidative stress.

Giménez-Siurana et al. employed a new drug delivery approach that incorporated RSV with silk fibroin nanoparticles (SFN) [19]. Silk fibroin (SF) has been shown to have an outstanding capacity to store a medication and serve as a vehicle for it in the form of nanoparticles [89,90,91]. This is particularly useful for overcoming the low bioavailability of RSV and its relatively rapid metabolism and elimination. Thus, in a DP rat model, Giménez-Siurana et al. compared the anti-inflammatory efficacy of liquid SF, RSV-SFN, and combination of both (SF + RSV-SFN). Treatment with RSV-SFN demonstrated the least inflammatory impact, whereas pairing it with liquid SF resulted in greatest reduction in inflammatory cytokines (IL-1β and IL-6) and thinnest epithelium [89,92]. This indicates that pairing the two might have a synergistic effect on inflammation in DP. Encapsulating polyphenols in SF nanoparticles has been shown to boost their capacity, improve their stability in the body, and prevent them from passing through unwanted metabolic processes [89].

Zare Javid et al. (2017) examined the anti-hyperglycemic and anti-inflammatory potential of RSV in clinical patients with DP [18]. Subjects with DP were randomized into two groups: intervention and control that received either RSV or placebo capsules, respectively, for four weeks along with non-surgical scaling and root planning (SRP). Clinically, supplementation resulted in significantly reduced periodontal pockets (PD) and better insulin levels and insulin resistance compared to controls, which has been described in previous studies [88,93]. Akin to earlier studies [93,94], RSV resulted in improved fasting blood glucose levels, however the difference was not significant when compared to the controls.

After 1 month of RSV supplementation, the same group reported a substantial reduction in inflammation, as measured by lower IL-6 levels, compared to the baseline [17]. Although this study demonstrated an insignificant improvement in total anti-oxidant capacity (TAC) in the test group, another study revealed otherwise [95]. A considerable reduction was observed in normocholesterolemic (NCs) individuals [95] and healthy subjects [96]. These discoveries suggest that RSV may improve TAC in healthy humans, but not in patients. In terms of periodontal status, CAL was examined and considerable improvements were observed in both groups; however, RSV supplementation alone did not significantly enhance the periodontal condition. CAL is a more precise metric since it uses a fixed reference point, typically the cemento-enamel junction (CEJ).

The investigations conducted on cell cultures and animal models show promising results for the effects of RSV in treating DP. The anti-diabetic, anti-inflammatory, and anti-oxidative potential of RSV seems to be enhanced when used in conjunction with diabetes medication (e.g., insulin) or delivered via nanoparticles (e.g., silk fibroin). Although the pre-clinical studies demonstrated remarkable outcomes, the limited clinical trials did not provide promising results. RSV in adjunct to SRP significantly resolved inflammation and improved PD, however it did not significantly alter fasting blood glucose, TAC, or CAL, which is a more accurate measure for periodontal loss around a tooth. This could be due to low bioavailability of RSV following oral administration and its quick metabolism and elimination. Thus, future research is crucial to improve the formulation and delivery system for RSV.

### 2.2. Curcumin

Curcumin (CUR) has been extensively studied these recent years for its therapeutic potential to modulate the immune system in DP [97]. It is a diferuloylmethane derived from the yellow spice turmeric, which is derived from the plant *Curcuma longa*. It has been reported as an efficient anti-oxidant, protecting numerous organs from oxidative stress-induced pathophysiology, including DP [23,24,98]. One clinical study and five pre-clinical studies were included in this review, as represented in Table 3.

This review identified three animal studies that evaluated the effect of a chemically modified curcumin (CMC2.24) on DP [22,23,24]. CMC2.24 is a phenylamino carbonyl curcumin, which is tri-ketonic in contrast to di-ketonic natural curcumin compounds. The advantages of this compound over the natural CUR includes greater efficacy as an MMP inhibitor and improved bioavailability. There was a consensus among the three studies, confirming the impact of CMC2.24 on bone loss reduction and inflammatory markers, where the activity of MMPs and the levels of pro-inflammatory cytokines were inhibited [22,23,24]. The outcomes of the mentioned studies confirmed that administration of CMC2.24 has an anti-inflammatory effect, and suggests it as an efficacious treatment for reducing bone resorption.

In 2016, Elburki et al. demonstrated the efficacy of CMC2.24 on DP rats, where they found a substantial reduction in alveolar bone loss and remarkable suppression of the pathological release of MMP-2, MMP-8, MMP-9, IL-6, and IL-1β [24]. It is well recognized that MMPs are responsible for the destruction of various constituents of the extracellular matrix, including collagen. In the following year (2017), the inhibitory effect of CMC2.24 on bone loss was shown to be mediated via the suppression of p38 MAPK and NF-κB signaling. Interestingly, CMC2.24 has been shown to be effective in DP in the presence of continuing severe hyperglycemia [99,100,101,102].

Likewise, Deng et al. demonstrated that CMC2.24 exerted no influence on glycemic control, however it was still able to "normalize" inflammatory and other cells. The compound reduced abnormal macrophage accumulation, improved defective chemotactic activity, and significantly enhanced resolvin (RvD1) activity [22]. The increased activity of RvD1 following CMC2.24 intervention was also reported in an earlier study [103]. RvD1 belongs to a group of molecules derived from omega-3 fatty acids that can orchestrate the resolution of acute inflammation [104]. These results suggest that CMC2.24 has no effect on blood glucose levels (at least in the short term), but it can target the complications of uncontrolled hyperglycemia involving an inflammatory/immune response that is exacerbated by host-microbial interactions.

Unlike CMC2.24, natural CUR possesses the ability to improve blood glucose levels. When Pimentel et al. compared the effect of natural CUR and insulin on combating DP, both had a similar impact on blood glucose [27]. When CUR and insulin were used in synergy, the improvements were superior in terms of alveolar bone level, glycemic control, and pro-inflammatory cytokine levels (IL-6 and IL-1β). Natural CUR was also found to significantly up-regulate RUNX2, a gene involved in the development and maintenance of the teeth, bones, and cartilage. Furthermore, CUR encouraged the up-regulation of SIRT [105], a protein that is persistently found reduced in DM and is associated with the regulation of mitochondrial anti-oxidant defense [106]. Moreover, when CUR was combined with insulin, the ratio of receptor activator by NF-κB ligand (RANKL)/OPG was decreased. The anti-inflammatory/oxidative activity of CUR and its modulation of RANKL/RANK/OPG expression may play crucial roles in delaying osteoclastogenesis and decreasing alveolar bone loss [107].

The anti-oxidative ability of CUR was further described in an animal investigation by Iova et al. [26]. CUR was shown to reduce oxidative stress, both locally and systemically, and boosted anti-oxidant enzymes, such as glutathione (GSH), oxidized glutathione (GSSG), GSH/GSSG, and catalase (CAT). These anti-oxidant enzymes are renowned for their ability to reduce oxidizing species, such as superoxide, peroxyl, and hydroxyl radicals [108,109].

In a clinical trial carried out by Ivanaga et al. [25], the clinical efficacy of CUR solution as a photo-sensitizer was evaluated for treating residual pockets in DP patients. After 6 months, it was found that LED irradiation alone or with CUR solution as an adjunct therapy to SR, provided a short-term (3 months) clinical benefit in terms of CAL gain, however there were no significant differences between the groups.

CMC2.24 and natural CUR each combat alveolar bone loss in DP in a slightly different way. CMC2.24 targets the hyper-immune/inflammatory response via suppression of MMPs, pro-inflammatory cytokines, and restoration of impaired immune cells while exerting no effect on blood glucose levels (at least in the short term) [22,23,24]. On the other hand, natural CUR repressed uncontrolled hyperglycemia, increased anti-oxidant capacity, and up-regulated anti-inflammation [25,26,27]. Caution must be taken when taking CUR together with anti-diabetic drugs to prevent hypoglycemia. Despite the hopeful results, toxicity and weight loss, as well as other negative consequences, were not observed in any of the trials considered, and the advantages of higher/lower dosages were not established. Different animal models are being used to conduct pharmacokinetic and safety research, which are required before this chemical can be tested in human clinical trials.

### 2.3. Melatonin

Melatonin‘s primary role is to regulate the sleep cycle in a circadian manner [110]. It is a common prescription sleep medication to regulate sleep. Melatonin (MLT) can be sourced from many plants, including Feverfew (*Tanacetumparthenium*) and St John’s wort (*Hypericumperforatum*) [111]. Interestingly, few studies have associated poor sleep quality with an increased risk of diabetes or a worsening of the condition [112,113], so it is worth looking into whether the effect of MLT in DP is attributable to better sleep quality. During the last decades, MLT was discovered as a noteworthy free radical scavenger [114,115,116] and has anti-inflammatory and glucose-regulatory properties [117,118]. As listed in Table 4, this review identified five clinical studies, two of which utilized topical application of MLT in cross-sectional studies, whereas the others utilized oral intake of the compound in clinical trials.

In the cross-sectional studies by Cutando et al. [31,32], DP patients were given topical MLT (1% orobase cream formula) in both upper and lower arches on the attached gingival surface. After 20 days of application, the results were measured in terms of clinical and biological parameters. Topical MLT significantly decreased the gingival index and pocket depth as well as serum IL-6 and CRP levels. Apart from its remarkable anti-inflammatory potential, topical MLT also down-regulated salivary RANKL and elevated salivary OPG levels, which is consistent with earlier studies [119,120,121]. The present data implies that RANKL is a key cytokine in bone destruction during periodontitis, and that OPG, as a RANKL decoy receptor, protects against destructive bone disease [122,123]. In light of this, MLT may have a favorable impact in slowing osteoclastogenesis, improving alveolar bone quality, and preventing the development of periodontal disease.

On the other hand, oral intake of MLT alongside SRP in investigations by Anton et al. [28] and Bazyar et al. [30] demonstrated an anti-inflammatory phenotype characterized by significantly improved PD and CAL, and repression of pro-inflammatory cytokines, such as IL-6, hs-CRP, and IL-1β. Moreover, MLT appears to have beneficial glucose-metabolic effects, since patients exhibited lower HbA1c after taking 3 mg of MLT (lower dosage) daily for 8 weeks. MLT consumption has been linked to improved glycemic management [124], and its use during the daytime has been linked to lower insulin sensitivity in other investigations [125,126,127].

Zare Javid et al. showed that ingestion of a higher dose of MLT (6 mg) daily for 8 weeks resulted in superior improvement for three anti-oxidant enzymes (SOD, CAT and GPX) and a lipid peroxidation marker (MDA) compared to the placebo group [29]. Previous studies demonstrated doses of 2 mg and 5 mg of MLT daily did not up-regulate these anti-oxidant enzymes, but both dosages significantly decreased MDA [128]. Since MDA is a marker of oxidative stress [129], this suggests that lower doses of MLT can exert anti-oxidant properties regardless of their effect on anti-oxidant enzymes. More research is needed to determine if the effect of MLT-induced overexpression of all three anti-oxidant enzymes on the anti-oxidant defense system is more or less effective as lower doses of MLT. If the higher doses are no better, then prescribing a lower dose of MLT is recommended.

From the clinical trials results, topical application of MLT for 20 days shows remarkable inflammation suppression, both clinically and biologically [31,32]. It is able to modulate local levels of pro-inflammatory cytokines and the RANKL/OPG ratio to slow osteoclastogenesis and deter the progression of DP. As for systemic intake of the supplement, MLT serves as a potent anti-oxidant [130] that is protective against oxidative stress [131] in periodontal tissue with clinical effects. Although MLT has been reported to modulate glucose-metabolic effects [132], it is still crucial that DM patients consult a physician beforehand to prevent excessive hypoglycemia when paired with anti-diabetic medication. Currently, there is no official recommended maximum dose for MLT in adults, but a range of 1 mg to 6 mg appears to be safe and effective [133]. Therefore, a low dose of MLT (under 6 mg) provides a therapeutic benefit as an adjunct to periodontal treatment for DP.

### 2.4. Propolis

Propolis (PP) is an organic resinous structure made by honeybees from a range of substances, such as plant parts, buds, and sap. The content of PP varies based on the plant source, which differs depending on the geographic zone, such as Brazil, Peru, Taiwan, and Europe. The biological and pharmacological capabilities of PP, which include anti-inflammatory, anti-bacterial [134], anti-oxidant [134,135], immunomodulatory actions [136], and anti-diabetic and anti-hyperglycemic properties [137], have sparked considerable interest. Recently, caffeic acid phenethyl ester (CAPE) has been discovered to be an important active molecule in PP and is responsible for most of its therapeutic properties [138].

To date, there have been two pre-clinical and one clinical study on the use of PP in DP, as summarized in Table 5. Aral et al. administered PP in DP mice models and showed that PP managed to reduce elevated blood glucose levels and repress alveolar bone loss, albeit with an insignificant decrease in pro-inflammatory cytokines [35]. In the study by Kızıldağ et al., the authors used caffeic acid phenethyl ester (CAPE) and investigated its effects in DP mice [34]. The exceptional anti-oxidative potential of CAPE was proven by up-regulated total anti-oxidant status as well as lowered total oxidant status and oxidative stress, comparable to a previous work [139]. Histochemistry analysis of the periodontal tissue demonstrated high scores for inflammatory reactions, ulcers, hyperemia, and the density of RANKL-stained cells, which were all alleviated after 15 days of CAPE intake. A high RANKL:OPG ratio has been implicated in the high rate of osteoclastogenesis and alveolar bone resorption during the course of periodontitis [140,141]. In line with previous evidence [142], it is plausible to suggest that CAPE exhibits the ability to inhibit ROS production, oxidative stress, and RANKL-induced osteoclastogenesis as it accelerates bone healing.

Vo et al. studied the influence of Taiwanese green propolis (TGP) on human gingival fibroblasts, and it was found to alleviate inflammation induced by high glucose levels [143]. This attenuation of inflammation occurred via inhibition of the NLRP3 inflammasome and its inflammatory cascade, which has been reported in other pathologies, such as gouty arthritis [144]. Activation of the NLRP3 inflammasome is known to be pro-inflammatory because it activates caspase-1, which governs pro-IL-1β maturation [145,146]. TGP targeted the upstream regulatory body involved in NLRP3 activation, including the NF-κB signaling pathway, Toll-like receptors (TLRs), and ROS production to protect gingival fibroblasts from inflammation.

Outcomes from the pre-clinical studies were comparable to those of the clinical study [33,143]. When DP patients were administered 400 mg PP daily for 6 months along with SRP treatment, glycemic control improved remarkably at both 3 and 6 months [33]. The clinical efficacy of PP was proven by significant improvements in PD and CAL in DP patients. Overall, the results for the long-term effectiveness of PP as an anti-diabetic, anti-inflammatory, and anti-oxidant for treating DP are promising. However, special precautions should be taken by patients who are allergic to bees or bee product, such as honey and conifers, since it may trigger allergic reactions.

### 2.5. Vitamin C

Vitamin C (Vit. C) has long been known as an essential micronutrient for humans that can be obtained from various fruits and vegetables, such as guavas, lemons, and broccoli. In subjects with DM, plasma Vit. C was significantly lower compared to healthy individuals [147]. Vit. C, which belongs to the group of free radical-scavenging anti-oxidants, promotes wound healing [148,149] and its supplementation in DP patients has been of interest lately.

This review included one animal study and two clinical trials, as demonstrated in Table 6. According to Toraman et al., local Vit. C treatment in DP rats significantly repressed alveolar bone loss (ABL) along with reduced MMP-8 and 8-OHdG in tissues, and serum CTX [36]. MMP-8 is known for its involvement in ECM breakdown, which can contribute to the severity of periodontitis [150], and 8-hydroxy2-deoxyguanosine (8-OHdG) is a critical biomarker of oxidative stress. Considering Vit. C has been shown to inhibit MMPs in other cells [151], it was proposed that Vit. C might help in suppressing oxidative stress and inflammation during DP, resulting in decreased bone resorption.

When Vit. C (450 mg) was given as a supplement to patients with DP for 2 weeks along with SRP, the bleeding index improved significantly compared to controls, despite unchanged PD and PI [38]. Similar outcomes have been described in non-smoker patients with periodontitis [136,152]. Although there were no changes in the PD and PI, it is nevertheless considered a slight clinical success since a decrease in bleeding tendency indicates the resolution of inflammation in the gingiva at least. When the dosage and period of Vit. C was increased to 500 mg for 2 months, no additional benefits were observed [37]. Plasma Vit. C was significantly increased in the intervention group, however there were no significant differences in periodontal parameters between the treatment and placebo groups, although they both improved from the baseline.

Vit. C did not alter FBS and HbA1c in DP patients, similar to another report [153]. It was reported that a higher dose of Vit. C (1000 mg) could elicit significantly decrease blood glucose [154]. Cellular uptake of vitamin C is promoted by insulin and inhibited by hyperglycemia [155]. This explains why higher doses of ascorbic acid might be necessary to overcome the effect of hyperglycemia to exert a beneficial effect.

In conclusion, supplementing initial periodontal treatment with 500 mg of Vit. C per day did not improve the periodontal state in periodontitis patients with uncontrolled type 2 diabetes. However, given the promising outcome for local Vit. C in the DP mouse model, and considering the challenge of cellular uptake of systemic Vit. C during hyperglycemia [36], it is worth investigating the effect of topical Vit. C in DP patients. Moreover, local injections of Vit. C have been shown to be an effective adjunctive treatment for reducing gingival inflammation in DP rats [156].

### 2.6. Green Tea

Green tea (GT) is known for its anti-oxidative [157], anti-bacterial [158], anti-inflammatory [158], and anti-diabetic [159] abilities. GT is a popular beverage across the world. It is often brewed from the dried leaves of Camellia sinensis, and the majority of its polyphenols are catechins, such as (-)-epicatechin (EC), (-)-epicatechingallate (ECG), (-)-epigallocatechin (EGC), and (-)-epigallocatechin 3-gallate (EGCG) [160,161].

Table 7 summarized the use of GT in DP. To date, there are one animal study and two clinical studies of GT in DP. In an early clinical trial (2013), Gadagi et al. used GT strips combined with supragingival scaling in periodontitis patients, both diabetic and non-diabetic, and evaluated its microbiological and clinical efficacy [39]. With hydroxypropyl methylcellulose (HPMC) as a vector, the GT was slowly released to the surrounding tissue, with full release occurring after 2 h. As a result, the clinical parameters, including GI, PD and CAL, improved in both groups (DM and non-DM) with GT intervention. At the same time, P. gingivalis was reduced at GT sites in non-DM patients (significantly) and DM patients (not significantly), according to its anti-microbial impact.

Apart from its anti-bacterial properties, with consistent clinical outcomes of the above study [39], some studies have observed that GT could return the levels of pro-inflammatory cytokines, TNF-α [162,163] and RANKL, [164] to normal while raising the expression of IL-10, RUNX-2, and OPG [41]. The down-regulation of pro-inflammatory cytokines coupled with up-regulation of IL-10 demonstrates that GT is a powerful anti-oxidant. This potential has been associated with suppression of the RANK system, synthesis of the anti-osteoclastogenic factor OPG, and a slowing down of resorption activity in the jawbone [165,166].

Catanzaro et al. demonstrated that giving DP mice GT improved their glycemic control, reduced plaque accumulation, and prevented periodontal disease progression [40]. Distinct from other studies [39,41], the authors investigated the effect of GT on the modulation of vascularization during the progression of DP. Chronic hyperglycemia is known to promote microvascular damage in the periodontium and this alteration has been associated with greater periodontal tissue loss in DM rats [167]. In individuals with microvascular complications, elevated VEGF expression has been found systemically [168,169]. The consumption of GT successfully inhibited microvascular alteration and returned the over-expression of VEGF to normal levels in DP mice.

In a nutshell, GT possesses anti-hyperglycemia, anti-inflammatory, anti-osteoclastogenic, and anti-oxidative effects in addition to inhibiting microvascular damage in periodontal tissue. One of the limitations of the animal studies is that the concentration of GT (which was brewed in water) in the animal models was not stated, since it may be challenging to regulate the volume of consumption by each rat. Be that as it may, the clinical trial showed promising results for locally applied GT in combating DP, and more studies should be carried out to enhance or extend the release of GT into periodontal tissue.

### 2.7. Ginger

Ginger is the rhizome of the plant *Zingiberofficinale Roscoe* and is one of the most widely used spice/herb worldwide [170]. Its major components, shogaol and gingerol, have demonstrated many beneficial pharmacological uses, including anti-diabetic, anti-oxidant, anti-inflammatory, anti-microbial, and anti-cancer [171]. Table 8 tabulated the research on the use of ginger in DP models.

When patients with mild to moderate DP were given 2 g of ginger daily for 8 weeks with SRP therapy, the patients demonstrated substantial clinical and biochemical inflammation resolution compared to the placebo group [42]. There were significant reductions in CAL and PD as well as decrements in inflammatory markers, such as IL-6, hs-CRP, and TNF-α. Likewise, Rehman et al. showed that zingerone therapy significantly decreased ROS levels, blocked nuclear factor-kappa B (NF-κB) activation, and reduced the levels of other inflammatory markers, including TNF-α, IL-6, and IL-1β in type 2 diabetic Wistar rat [172]. Apart from its anti-inflammatory effect, 6-shogaol has been reported to up-regulate the expression of anti-oxidants such as GPx and SPD in a clinical trial [42] as well as in animal models [173]. These findings indicate that ginger may protect DM patients from oxidative damage by decreasing oxidative stress and minimizing anti-oxidant depletion [174]. In a study of its molecular mechanism, 6-shogaol, the most abundant component in shogaol extract, demonstrated powerful anti-oxidative capacity by regulating oxidative and anti-oxidative activities in human gingival fibroblasts (HGF) [175]. In addition, 6-shogaol was found to inhibit ROS production [176], increase levels of HO-1 [177] and NQO1, and ameliorate the inflammatory cascade [178,179].

Overall, the anti-inflammatory and anti-oxidative abilities of ginger supplementation may be more effective for the control of systemic inflammation in DP patients compared to SRP alone, at least for a short period (2 months). It is recommended that future studies include post-intervention glycemic status to assess the anti-diabetic capacity of ginger and for any side effects, especially when paired with medications that lower blood glucose in DM patients.

### 2.8. Others: Aloe Vera, Rutin, Vitamin E

#### 2.8.1. Aloe Vera

The research on the use of aloe vera (AV), rutin (RT), and vitamin E (Vit. E) in DP were listed in Table 9. Aloe vera (*Aloe barbadensis Miller*), also known as Ghritkumari/Gwarpatha, is a member of the Liliaceae family and has been utilized in traditional medicines for centuries. Aloe vera (AV) is well-known for its various therapeutic properties, for example, anti-inflammatory [180], anti-oxidant [181], anti-bacterial [182], and immune boosting properties. Apart from that, it has been shown in diabetic individuals to exhibit hypoglycemic and hypolipidemic effects [183]. Only one clinical study has assessed the effectiveness of AV in DP prevention. Pradeep et al. delivered AV in gel form to the periodontal pocket of DP subjects and measured its clinical effectiveness 3- and 6-months post-treatment [43]. When compared to the control group, the intervention group showed greater improvements in PI, mSBI at 3 months, and significant CAL gain at 6 months. This improvement might be explained by the antibacterial action of AV against periodontal infections, which has been observed in various investigations [182,184]. When used as an adjunct to SRP, local application of AV gel can benefit DP patients in terms of both immediate and long-term clinical efficacy, even in moderate to severe cases. To corroborate the findings of this investigation, long-term, double-blind, multicenter randomized clinical studies are required, and should include glycemic state assessment, histological bone gain evaluation, and microbiologic profiles.

#### 2.8.2. Rutin

Rutin, a citrus flavonoid glycoside found in fruits such as orange, grapefruit, lemon, apples, and berries is a common element in multivitamin and herbal cures [185]. This complex has a wide variety of medicinal purposes, including anticancer, antibacterial, and anti-inflammatory effects [186,187]. Furthermore, rutin has been found to be effective in diabetes and malignancies [188]. Rutin, alone or in combination with curcumin, has been proven to reduce malondialdehyde (MDA), an oxidative stress biomarker, in gingiva tissue and blood when compared to rats without therapy. Moreover, rutin significantly enhanced anti-oxidant enzymes, including glutathione (GSH), oxidized glutathione (GSSG), GSH/GSSG, and catalase (CAT). These enzymes possess reducing characteristics for various oxidizing species, such as peroxyl, superoxide, and hydroxyl radicals [189]. Although it has been established that increased MDA levels and reduced GSH levels in diseased periodontal tissue correlate with more severe alveolar bone loss, future research is needed to evaluate whether rutin can transfer its promising effects to DP patients.

#### 2.8.3. Vitamin E

Vitamin E is a fat-soluble vitamin, which consists of two main forms: α-tocopherols and γ tocopherols. The anti-oxidative properties of vitamin E have been well recognized along with its role in anti-inflammatory processes, and it has been implicated in preventing diseases such as cardiovascular diseases and cancer [190]. At present, vitamin E inhibited inducible nitric oxide synthase (iNOS) activity in a murine DP model. It was postulated that vitamin E represses gingival inflammation via lowering NO and hence oxidative stress, which agrees with a previous study on rats with periodontitis [191]. iNOS is a type of NO synthase present in macrophages [192,193] that releases nitric oxide (NO), a short-lived free radical [194]. iNOS expression has been shown to be higher in gingiva obtained from mice and patients with DP [195,196,197], however iNOS expression did not appear to have an additional deleterious effect on the clinical course of DP compared to periodontitis alone [197]. Due to the limited data concerning the pathogenic function of iNOS in the progression of DP, additional research is needed, particularly on vitamin E’s influence on periodontal and glycemic parameters in DP models to determine its clinical efficacy in combating DP.

## 3. Strengths, Limitations and Future Directions

By offering a broad review of pertinent research, this scoping study evaluated the existing knowledge of the issue of interest, noting trends, advancements, and comparability within and across each natural product. This review also enables us to identify gaps in the literature, such as the impact of different administration routes (topical vs. systemic) and doses on DP, as well as the uniformity of data collection methods. Additionally, our results may provide a platform for future research to contribute into the formulation of adjunct therapy using natural products in patients with DP. Nevertheless, this review has certain limitations, for example, to conclude a definitive effect of each natural bioactive on DP is nearly impossible due to the limited research data and the lack of homogeneity among study designs. Another factor is the small sample size in most randomized clinical trials (RCTs) with short follow-up period. 

Considering the shortcomings of the present data mentioned in this review, future research might include the following suggestions. Clinical trials should include: (1) detailed descriptions of inclusion and exclusion criteria, case definition of DM, severity of periodontitis, etc.; (2) bigger sample sizes and repeated HbA1c to minimize inaccuracy; (3) longer follow-up to assess the sustainability of the treatment’s impact and any unwanted effects; (4) emphasis on designing a double-blind studies to reduce any bias risk. As for animal studies, they should include: (1) accurately described methods for the induction of either DM type 1 or 2; (2) measure accelerated periodontitis in animals (e.g., rats) that are extremely resistant to periodontal diseases under normal circumstances including ligature fixation, bacteria inoculation, etc. [198].

## 4. Conclusions

Usage of natural products has been practiced since ancient times and has continued in the current civilization for well-being and treatment for various disorders. Over the past decade, natural compounds have emerged as an adjunct approach for modulating the host immune response to resolve diabetes-associated periodontitis. Our paper has reviewed the data from various studies on the subject, focusing on the therapeutic benefits of natural compounds for diabetes-associated periodontitis. According to the findings, the mentioned natural compounds have a variety of positive pharmacological qualities, such as anti-oxidative, anti-inflammatory, anti-osteoclastogenesis, and anti-bacterial. Since these natural compounds possess medicinal potential for curbing the hyperinflammatory components of the host response, they might serve as novel adjunct remedies for controlling diabetes-associated periodontitis. However, more study is needed to maximize their therapeutic effect as well as determining the amount of natural extract in the final product, which can then be evaluated in well-designed clinical trials.

## Figures and Tables

**Table 1:** Summary of study types for each natural compound and its clinical evaluation.

Type of Natural Products	Studies Type Included		Applicable to Clinical Studies Only				
			Route of Treatment		Dosage	Improvements in:	
	ANI	CLIN	TOP	SYS		Periodontitis (PD/CAL/ABL)	DM (FBG/HbA1c/Insulin Resistance)
Resveratrol	/	/	-	/	480 mg/day- 30 days	/	/
Curcumin	/	/	/	-	100 m/L as irrigant- single session	/	N.A.
CMC2.24	/	-	N.A.		N.A.	N.A.	N.A.
Melatonin	-	/	/	/	Top. cream 1%- 20 days	/	-
					Syst-3–6 mg/day- 8 weeks	/	/
Propolis	/	/	-	/	400 mg/day- 6 months	/	/
Vitamin C	/	/	-	/	450–500 mg/day– 2 weeks–2 months	-	-
Green Tea	/	/	/	-	0.204 g strip- single session	/	N.A.
Ginger	-	/	-	/	2 g/day- 8 weeks	/	N.A.
Aloe Vera	-	/	/	-	Top. gel-N.A. dose single session	/	N.A.
Rutin	/	-	N.A.		N.A.	N.A.	N.A.
Vitamin E	/		N.A.		N.A.	N.A.	N.A.

ABL, alveolar bone loss; ANI, animal study; CAL, clinical attachment loss; CLIN, clinical study; FBG, fasting blood glucose; HbA1c, hemoglobin A1c; N.A., not available; PD, pocket depth; SYS, systemic; TOP, topical.

**Table 2:** Studies on the use of resveratrol (RSV) in diabetes-associated periodontitis (DP).

Author (Year)	Types of Study	Dosage & Administration Route	Treatment Duration	Study vs. Control Groups	Study Outcomes
Zare Javid et al. (2019) [17]	CLIN (chronic P: moderate)	480 mg/day RSV orally	30 days	(1) SRP + Placebo (2) SRP + RSV	▪ Both groups ↑ CAL gain, but no significant difference between them. ▪ RSV ↓ IL-6, ↑ TAC.
Zare Javid et al. (2017) [18]	CLIN (chronic P: all stages)	480 mg/day RSV orally	30 days	(1) SRP + Placebo (2) SRP + RSV	▪ RSV improved insulin level, insulin resistance and PD. ▪ RSV ⬌ made to FBG and TG.
Giménez- Siurana et al. (2019) [19]	ANI (rats, STZ-induced DM; 50 mg/kg single dose)	3 mg/day RSV-SFN via oral gavage	30 days	(1) CMC (2) CMC + SF (3) CMC + RSV-SFN (4) CMC + SF + RSV-SFN (5) Water	▪ Group 3: lowest area of inflammatory focus. ▪ Group 4: greatest drop in IL-1β, IL-6 and has lowest epithelium thickness. ▪ Overall, liquid SF + RSV-SFN work synergically in controlling DP.
Cirano et al. (2021) [20]	ANI (rats, STZ-induced DM; 60 mg/kg single dose)	10 mg/kg/day RSV via oral gavage	30 days	(1) DM + Placebo (2) DM + INS (3) DM + RSV (4) DM + RSV + INS (5) Non-DM	▪ RSV alone ↓ NADPH oxidase activity. ▪ RSV+ INS ↓ ABL and IL-1β, IL-6, IL-17, oxidative stress markers. ▪ RSV, alone or combined with insulin ↑ anti-oxidants, SOD and SIRT l in DP.
Zhen et al. (2015) [21]	ANI (DM type 2 genetic mice)	20 mg/kg/day RSV via oral gavage	30 days	(1) DM (2) DP (3) DP + RSV	▪ RSV ↓ FBG, ABL in DP. ▪ mRNA, protein levels of TLR4 ↑ in DP. ▪ RSV ↓ the inflammatory response via ↓ TLR4 expression and ↓ activation of TLR4 downstream signaling.
	IV (GEC of DM type 2 genetic mice)	10 µM RSV	24 h prior to stimulation	(1) Non-HG (2) HG (3) HG + RSV	

↑ up-regulate; ↓ down-regulate; ⬌ no change; ANI, animal study; CLIN, clinical study; ABL, alveolar bone loss; CAL, clinical attachment level; DM, diabetes mellitus; FBG, fasting blood glucose; GEC, gingival epithelial cells; HG, high glucose; IL, interleukin; INS, insulin; NADPH, nicotinamide adenine dinucleotide phosphate; PD, pocket depth; SIRT, silent information regulator; SOD 1, superoxide dismutase; TAC, total anti-oxidant capacity; TG, triglyceride; TLR, Toll-like receptor.

**Table 3:** Studies on the use of curcumin (CUR) in diabetes-associated periodontitis (DP).

Author (Year)	Types of Study	Dosage & Administration Route	Treatment Duration	Study vs. Control Groups	Study Outcomes
CMC2.24					
Deng et al. (2021) [22]	ANI (rats, STZ-induced DM; 70 mg/kg single dose)	30 mg/kg/day CMC2.24 via oral gavage	3 weeks	(1) DP (2) DP + CMC2.24	▪ CMC2.24 ⬌on BG, HbA1c. ▪ CMC2.24 ↓ ABL, DM-induced macrophage accumulation and impaired chemotactic activity. ▪ CMC2.24 is anti-inflammatory, ↓ MMP-9, pro-resolvin.
Elburki et al. (2017) [24]	ANI (rats, STZ-induced DM; 70 mg/kg single dose)	30 mg/kg/day CMC2.24 via oral gavage	3 weeks	(1) DP (2) DP + CMC2.24	▪ CMC 2.24 ↓ ABL and ↓ IL-1β, IL-6. ▪ CMC2.24 ↓ MMP-9, NF-κB (p65) and p38 MAPK.
Elburki et al. (2016) [23]	ANI (rats, STZ-induced DM; 70 mg/kg single dose, DM type 1)	30 mg/kg/day CMC2.24 via oral gavage	3 weeks	(1) DP (2) DP + CMC2.24	▪ CMC 2.24 has ⬌ on BG, ↓ ABL. ▪ CMC 2.24 ↓ IL-1β, IL-6 (x significant). ▪ CMC 2.24 ↓ MMPs to near normal levels.
CUR					
Ivanaga et al. (2019) [25]	CLIN (chronic P: all stages)	100 mg/L CUR solution via irrigation	Single session	(1) SRP (2) SRP + CUR (3) SRP + LED (4) SRP + CUR+ LED	▪ CUR alone and with LED irradiation to SRP ↑ PD gain at 3- and 6-months post-therapy. ▪ CUR + LED irradiation to SRP provide CAL gain at 3 months.
Iova et al. (2021) [26]	ANI (rats, STZ-induced DM; 30 mg/kg & 30% glucose single dose)	75 mg/kg/day CUR via oral gavage	10 weeks	(1) DP (2) DP + CUR (3) DP + Rutin (4) DP + CUR+ Rutin	▪ CUR, single or paired with Rutin, ↓ oxidative stress both in gingival tissue and blood. ▪ CUR ↑ anti-oxidant status in DP rats.
Pimentel et al. (2020) [27]	ANI (rats, STZ-induced DM; 60 mg/kg single dose)	100 mg/kg/day CUR via oral gavage	30 days	(1) DP + placebo (2) DP + CUR (3) DP + INS (4) DP + CURC + INS	▪ CUR ↓ BG, similar to INS effect. ▪ CUR ↑ Runx2 and SIRT gene. ▪ CUR + INS synergically ↓ BG, ABL. ▪ CUR +INS ↓ IL-6, IL-1β, RANKL/OPG ratio.

↑ up-regulate; ↓ down-regulate; ⬌ no change; ANI, animal study; CLIN, clinical study; ABL, alveolar bone loss; BG, blood glucose; CAL, clinical attachment level; CMC2.24, chemically modified curcumin; DM, diabetes mellitus; HbA1c, hemoglobin A1c; IL, interleukin; INS, insulin; MAPK, mitogen-activated protein kinase; MMPs, matrix metalloproteinases; PD, pocket depth; RANKL/OPG, receptor activator of nuclear factor-kappa B ligand/osteoprotegerin; SIRT, silent information regulator; sost, sclerostin; SRP, non-surgical scaling and root planning.

**Table 4:** Studies on the use of melatonin (MLT) in diabetes-associated periodontitis (DP).

Author (Year)	Types of Study	Dosage & Administration Route	Treatment Duration	Study vs. Control Groups	Study Outcomes
Anton et al. (2021) [28]	CLIN (chronic P: all stages)	3 mg/day MLT orally	8 weeks	(1) SRP + Placebo (2) SRP + MLT	▪ MLT improved PD, CAL and glycemic control. ▪ BOP, hygiene level improved more significant in MLT group.
Javid (2020) [29]	CLIN (chronic P: mild, moderate)	6 mg/day MLT orally	8 weeks	(1) SRP + Placebo (2) SRP + MLT	▪ MLT ↓ IL-1β and MDA. ▪ MLT ↑ serum levels of TAC, SOD, CAT, GPx.
Bazyar et al. (2019) [30]	CLIN (chronic P: mild, moderate)	6 mg/day MLT orally	8 weeks	(1) SRP + Placebo (2) SRP + MLT	▪ MLT ↑ serum MLT, ↓ IL-6 and hs-CRP and improved PD, CAL.
Cutando et al. (2015) [31]	CLIN (P: all stages)	Topical MLT (1% orabase cream formula)	20 days	(1) Placebo (2) MLT	▪ MLT ↓ GI, PD and ↓ IL-6 and CRP serum levels.
Cutando et al. (2014) [32]	CLIN (P: all stages)	Topical MLT (1% orabase cream formula)	20 days	(1) Placebo (2) MLT	▪ MLT ↓ GI, PD, salivary levels of RANKL, and ↑ salivary OPG.

↑ up-regulate; ↓ down-regulate; CLIN, clinical study; BOP, bleeding on probing; CAL, clinical attachment level; CAT, catalase; CRP, C-reactive protein; GPx, glutathione peroxidase; hs-CRP, high-sensitivity C-reactive protein; IL, interleukin; MDA, malondialdehyde; PD, pocket depth; RANKL, receptor activator of nuclear factor-kappa B ligand; SRP, non-surgical scaling and root planning.

**Table 5:** Studies on the use of propolis (PP) in diabetes-associated periodontitis (DP).

Author (Year)	Types of Study	Dosage & Administration Route	Treatment Duration	Study vs. Control Groups	Study Outcomes
El-Sharkaw et al. (2016) [33]	CLIN (chronic P: moderate to severe)	400 mg/day PP orally	6 months	(1) SRP (2) SRP + PP	▪ PP ↓ HbA1c level, FBG, CML both 3 and 6 months after SRP. ▪ PP improved PD, CAL at 3 and 6 months.
Kızıldağ et al. (2020) [34]	ANI (rats, STZ-induced DM; 50 mg/kg single dose)	10 mM/kg/day CAPE via oral gavage	15 days	(1) DP (2) DP + CAPE	▪ CAPE ↓ alveolar ABL, OSI and IL-1β. ▪ CAPE ↓ histochemistry score and RANKL-stained cells. ▪ CAPE ↓oxidative stress via ↑ TAS and ↓ TOS, OSI, RANKL and IL-1β levels.
Aral et al. (2015) [35]	ANI (rats, STZ-induced DM; 150 mg/kg single dose)	100 mg/kg/day PP orally	3 weeks	(1) DP (2) DP + PP	▪ PP ↓ BG, ABL in DP. ▪ PP ⬌ plasma IL-1β, TNF-α, MMP-8.

↑ up-regulate; ↓ down-regulate; ⬌ no change; ANI, animal study; CLIN, clinical study; ABL, alveolar bone loss; BG, blood glucose; CAPE, caffeic acid phenethyl ester; IL, interleukin; MMP-8, matrix metalloproteinase-8; OSI, oxidative stress index; P, periodontitis; RANKL, receptor activator of nuclear factor-kappa B ligand; TNF-α, tumor necrosis factor alpha.

**Table 6:** Studies on the use of vitamin C (Vit. C) in diabetes-associated periodontitis (DP).

Author (Year)	Types of Study	Dosage & Administration Route	Treatment Duration	Study vs. Control Groups	Study Outcomes
Toraman et al. (2020) [36]	ANI (rats, ALX- induced DM; 150 mg/kg single dose)	50 μLVit C injected to buccal gingiva	3 times with 2 days interval after ligature removal.	(1) DP + placebo (2) DP + Vit C	▪ Vit. C ↓ ABL and ↓ CTX, 8-OHdG, and AGE ↓ in the DP group
Kunsongkeit et al. (2019) [37]	CLIN (chronic P: moderate to severe)	500 mg/day Vit. C orally	2 months	(1) SRP + placebo (2) SRP + Vit. C	▪ Vit. C ↑ plasma vit. C, ⬌ FBG and HbA1c. ▪ All periodontal parameters were improved in both groups, but no significant difference between them.
Gokhale et al. (2013) [38]	CLIN (chronic P: moderate to severe)	450 mg of AA orally	2 weeks	(1) SRP + placebo (2) SRP + AA	▪ AAL plasma levels were ↓ in DP patients. ▪ Vit. C ↓ sulcus bleeding index in DP, ⬌ in PI, PD

↑ up-regulate; ↓ down-regulate; ⬌ no change; ANI, animal study; CLIN, clinical study; AA, ascorbic acid; ABL, alveolar bone loss; AGE, advanced glycation end-products; ALX, alloxan monohydrate; CTX, C-terminal telopeptide fragments; DM, diabetes mellitus; FBG, fasting blood glucose; HbA1c, hemoglobin A1c; P, periodontitis; PD, pocket depth; PI, plaque index; SRP, non-surgical scaling and root planing.

**Table 7:** Studies on the use of green tea (GT) in diabetes-associated periodontitis (DP).

Author (Year)	Types of Study	Dosage & Administration Route	Treatment Duration	Study vs. Control Groups	Study Outcomes
Gadagi et al. (2013) [39]	CLIN (chronic P: all stages)	Local delivery of 0.204 g of GT strips	Single session	(1) SRP + placebo (2) SRP + GT strips	▪ 10.67% of GT is released at first 30 min and ended at 120 min with full release. ▪ GT improved GI, PD and CAL.
Catanzaro et al. (2018) [40]	ANI (rats, STZ-induced DM; 47 mg/kg single dose, DM type 1)	7 g GT diluted in 1 L distilled water ad libitum	3 months	(1) water (2) GT	▪ GT ↓ FBG, ↓ DM-induced increased MVD, ↓ number of CD31 and VEGF-stained cells. ▪ GT ↓ dental plaque accumulation and severity of periodontitis.
Gennaro et al. (2015) [41]	ANI (rats, STZ; 50 mg/kg single dose, DM type 1)	7 g GT diluted in 1 L distilled water ad libitum	3 months	(1) water (2) GT	▪ GT ↓ TNF-α, RANKL. ▪ GT ↑ IL-10, RUNX-2 and OPG.

↑ up-regulate; ↓ down-regulate; ANI, animal study; CLIN, clinical study; CAL, clinical attachment level; DM, diabetes mellitus; FBG, fasting blood glucose; GI, gingival index; IL-10, interleukin-10; MVD, microvascular density; OPG, osteoprotegerin; P, periodontitis; PD, pocket depth; RANKL, receptor activator of nuclear factor-kappa B ligand; RUNX-2, Runt-related transcription factor 2; TNF-α, tumor necrosis factor alpha; VEGF, vascular endothelial growth factor.

**Table 8:** Studies on the use of ginger (GG) in diabetes-associated periodontitis (DP).

Author (Year)	Types of Study	Dosage & Administration Route	Treatment Duration	Study vs. Control Groups	Study Outcomes
Zare Javid et al. (2019) [42]	CLIN (chronic P: mild to moderate)	2 g/day ginger orally	8 weeks	SRP + placebo SRP + GG	▪ GG ↓ IL-6, hs-CRP, TNF-α, CAL, and PD. ▪ GG ↑ SOD and GPx, however SOD difference between groups was not significant.

↑ up-regulate; ↓ down-regulate; CLIN, clinical study; CAL, clinical attachment level; GPx, glutathione peroxidase; hs-CRP, high-sensitivity C-reactive protein; IL-6, interleukin-6; PD, pocket depth; SOD, superoxide dismutase; SRP, non-surgical scaling and root planing; TNF-α, tumor necrosis factor alpha.

**Table 9:** Studies on the use of aloe vera (AV), rutin (RT), and vitamin E (Vit. E) in diabetes-associated periodontitis (DP).

Author (Year)	Types of Study	Dosage & Administration Route	Treatment Duration	Study vs. Control Groups	Study Outcomes
Pradeep et al. (2015) [43]	CLIN (chronic P: moderate to severe)	AV gel (injected to periodontal pocket)	Single session	(1) SRP + placebo (2) SRP + AV gel	▪ AV group showed greater ↓ in PI, mSBI, and PD (3 months) ▪ AV ↑ CAL gain at 3 and 6 months.
Iova et al. (2021) [26]	ANI (rats, STZ-induced DM; 30 mg/kg & 30% glucose single dose)	75 mg/kg/day RT via oral gavage	10 weeks	(1) DP (2) DP + CUR (3) DP + Rutin (4) DP + CUR+ RT	▪ RT, single or combined with CUR, ↓ oxidative stress in gingival tissue and blood and ↑ anti-oxidant status.
Hatipoglu et al. (2016) [44]	ANI (rats, STZ-induced DM; 50 mg/kg single dose)	40 mg/kg/daily Vit. E (α-tocopherol) via intraperitoneal	3 weeks	(1) DP + saline (2) DP + α-tocopherol	▪ α-tocopherol↓ number of iNOS positive cells.

↑ up-regulate; ↓ down-regulate; ANI, animal study; CLIN, clinical study; CAL, clinical attachment level; CAT, catalase; CUR, curcumin; mSBI, modified sulcus bleeding index; PD, pocket depth; PI, plaque index.

## Data Availability

Not applicable.
